# Glycated Hemoglobin and Outcomes in Patients with Advanced Diabetic Chronic Kidney Disease

**DOI:** 10.1038/srep20028

**Published:** 2016-01-28

**Authors:** I-Ching Kuo, Hugo You-Hsien Lin, Sheng-Wen Niu, Daw-Yang Hwang, Jia-Jung Lee, Jer-Chia Tsai, Chi-Chih Hung, Shang-Jyh Hwang, Hung-Chun Chen

**Affiliations:** 1 Department of Internal Medicine, Kaohsiung Municipal Ta-Tung Hospital, Kaohsiung Medical University, Kaohsiung, Taiwan; 2 Graduate Institute of Medicine, College of Medicine, Kaohsiung Medical University, Kaohsiung, Taiwan; 3 Division of Nephrology, Department of Internal Medicine, Kaohsiung Medical University Hospital, Kaohsiung Medical University, Kaohsiung, Taiwan; 4 Faculty of Renal Care, College of Medicine, Kaohsiung Medical University, Kaohsiung, Taiwan; 5 Institute of Population Sciences, National Health Research Institutes, Miaoli, Taiwan

## Abstract

Diabetes is the major risk factor for end-stage renal disease (ESRD) worldwide. In advanced chronic kidney disease (CKD), less is known about the predictive value of HbA1c. We enrolled 2401 diabetic patients with stage 3–4 and stage 5 CKD, who were classified into 4 groups according to their baseline HbA1c values (<6%, 6%–7%, 7%–9%, and >9%). During the median follow-up of 3 years, 895 patients developed ESRD, and 530 died. In linear regression analysis, higher HbA1c correlated with higher eGFR in patients with stage 5 CKD but not in stage 3–4 CKD. In Cox regression analysis, a trend toward worse clinical outcomes existed when the HbA1c level exceeded 6% in stage 3–4 CKD, but the significance was only observed for >9%. The hazard ratios (HRs) for ESRD, all-cause mortality and combined CV events with mortality in the group of HbA1c >9% were 1.6 (95% CI, 1.07 to 2.38), 1.52 (95% CI, 0.97 to 2.38) and 1.46 (95% CI, 1.02 to 2.09), respectively. This study demonstrates that the higher HbA1c level is associated higher risks for clinical outcomes in diabetic patients with stage 3–4 CKD but not in stage 5 CKD.

Diabetes mellitus (DM) is the leading cause of chronic kidney disease (CKD) worldwide, accounting for approximately 45% of end-stage renal disease (ESRD) cases in the Taiwan dialysis population. Measuring glycated hemoglobin (HbA1c) has been suggested as a means of assessing glycemic control in patients with diabetes. Current guidelines recommend a target HbA1c of approximately 7% for preventing or delaying microvascular complications, including diabetic kidney disease^1^,^2^. Furthermore, several randomized controlled trials of patients with type 2 DM and preserved kidney function have demonstrated that tight glycemic control targeting a HbA1c level of <6%–6.5% reduced the development and progression of albuminuria, but the effect on specific renal end points, including ESRD, was inconclusive^3^,^4^,^5^,^6^. However, a meta-analysis of randomized controlled trials illustrated that intensive glucose lowering might reduce nonfatal coronary events, but a discrepancy remained regarding its benefits on all-cause mortality^7^.

Less is known regarding how glycemic control affects clinical prognosis in patients with DM and in later stages of CKD, whom were mostly excluded from clinical trials. Two major problems are encountered in these patients. First, HbA1c might not be an effective indicator of glycemic control and thus not a good predictor of patient prognoses. Second, glycemic control to lower HbA1c targets might be related to hypoglycemia occurrences. One cohort study demonstrated that, in diabetic patients with stage 3–4 CKD, higher (>9%) and lower (<6.5%) HbA1c levels both appeared to associate with poorer clinical outcomes regardless of the baseline estimated glomerular filtration rate (eGFR)^8^. Another study showed that, in dialysis-dependent people with DM, patients with higher HbA1c levels, particularly those without anemia, exhibited poorer survival rates than did patients in the HbA1c range of 5%–6%^9^. To elucidate these equivocal results, we analyzed the relationships between HbA1c and the risks of ESRD and mortality in the advanced stages of diabetic CKD and tested whether different CKD stages affected these relationships.

## Methods

### Participants and Measurements

This was an observational study that enrolled patients with CKD who were treated as part of the integrated or traditional care program of 2 affiliated hospitals of Kaohsiung Medical University in Southern Taiwan. The study was conducted from November 11, 2002 to May 31, 2009, with follow-up until May 31, 2010. We excluded patients who had a record of acute kidney injury, defined as a more than 50% decrease in the eGFR within 3 months, or had received chronic renal replacement therapy (RRT) before their first visit. The cohort comprised 4824 patients, and we selected 2401 patients with stage 3–5 CKD and type 2 DM as defined by the World Health Organization for this study^10^. CKD stages were defined as follows: stage 3, eGFR of 30 to 59 mL/min/1.73 m2; stage 4, eGFR of 15 to 29 mL/min/1.73 m2; and stage 5, eGFR less than 15 mL/min/1.73 m2 based on staging criteria from the National Kidney Foundation Kidney Disease Outcomes Quality Initiative (KDOQI)^11^. All participants were followed at clinic visits periodically for routine biochemical blood exams and evaluation of CKD complications. The institutional review board of Kaohsiung Medical University Hospital approved the study protocol, and informed consent was obtained from all participants. The methods were carried out in accordance with the Declaration of Helsinki ethical principles for medical research.

Participant demographic information was gathered upon their first visit, and their medical histories were obtained using a chart review. Their baseline biochemical data and comorbidities were analyzed. The eGFR of the participants was calculated using the simplified modification of diet in renal disease (MDRD) study equation: eGFR mL s-1 [1.73m2]-1 = 186 × serum creatinine −1.154 × age −0.203 × 0.742 (if female) × 1.212 (if black). In Taiwan, the MDRD formula was applied in the Taiwan National Database to evaluate CKD prevalence and dialysis initiation^12^,^13^. Therefore, we chose MDRD formula over CKD-EPI (Epidemiology Collaboration) as our study equation. The HbA1c value was measured as clinically indicated by the hospital laboratory using automated cation-exchange high-performance liquid chromatography. There was no substantial change to the HgbA1c measurement methodology during the study.

The patients were classified into 4 groups according to the following thresholds, which were selected according to guidelines and clinical trials, depending on their first HbA1c measurement: <6%, 6%–7%, 7%–9%, and >9%. The participants were diagnosed with hypertension if their office blood pressure was >140/90 mmHg or if they took any antihypertensive medications. Cardiovascular (CV) diseases were defined as clinically diagnosed myocardial infarction, heart failure, ischemic heart disease, and cerebrovascular disease. The first measurements of laboratory data, namely the urine protein-to-creatinine ratio (UPCR), albumin, hemoglobin, blood glucose, HbA1c, total calcium, phosphate, uric acid, total cholesterol, triglyceride, and C-reactive protein (CRP), were used as baseline variables.

### Outcomes

The primary outcomes of this study were ESRD, CV events, and all-cause mortality. ESRD was defined as the initiation of hemodialysis, peritoneal dialysis, or renal transplantation. The development of ESRD was ascertained using catastrophic illness cards issued by the Bureau of National Health Insurance. CV events were confirmed by examining records of hospitalization with the responsible diagnosis for acute coronary syndrome (International Classification of Diseases, Ninth Revision, Clinical Modification: 410.x–412.x), acute cerebrovascular disease (430.x–438.x), and congestive heart failure (428.x) or death from the aforementioned causes, but only in patients with CV event occurrence after the index date. All-cause mortality was determined using death certificates and the National Death Index. Hypoglycemia was defined as the presence of typical symptoms and signs of hypoglycemia requiring medical assistance.

### Statistical Analysis

Descriptive statistics were expressed as counts and percentages for the categorical data, and means with standard deviations or medians with interquartile ranges were determined for continuous variables with approximately normal distributions. Differences in baseline characteristics between groups were analyzed using ANOVA tests for continuous variables and chi-squared tests for categorical variables. For skewed distributions of some continuous variables, we applied logarithmic transformation to make data conform more closely to the normal distribution (cholesterol and CRP). We used multivariate linear regression analysis to investigate possible individual related variables, with the HbA1c level as the dependent variable. To determine the relative associations between the baseline HbA1c level and clinical outcomes, a Cox multivariate regression model was employed and adjusted for age, sex, the eGFR, the log-transformed UPCR, CV disease, mean blood pressure, hemoglobin, albumin, log-transformed cholesterol, log-transformed CRP, phosphorus, and the body mass index. Covariates were selected on the basis of their significance in statistics or on the basis of clinical relevance^14^. Furthermore, we performed subsequent subgroup analysis to observe the relationship of HbA1c with clinical outcomes among these demographic, clinical, and laboratory categories. A *P* value of <0.05 was considered statistically significant. All analyses were conducted using R 2.15.2 software (R Foundation for Statistical Computing, Vienna, Austria) and the Statistical Package for Social Sciences for Windows Version 18.0 (SPSS Inc., Chicago, IL).

## Results

### Baseline Characteristics by HbA1c

Table 1 summarizes the baseline characteristics of the 2401 patients with DM and stage 3–4 and stage 5 CKD classified according to HbA1c level. The cohort had a mean age of 64.3 ± 12.6 years, mean eGFR of 24.7 ± 14.9 ml/min/1.73 m^2^, medium UPCR of 1738 (547–4547) mg/g, mean hemoglobin of 10.7 ± 2.3 g/dl, and mean HbA1c of 7.4 ± 1.7%. Of the 1558 patients with stage 3–4 CKD, those with a HbA1c level of <6%, 6%–7%, 7–9%, and >9% comprised 16.8%, 28.7%, 37.9%, and 16.6%, respectively. Of the 843 patients with stage 5 CKD, those with a HbA1c level of <6%, 6%–7%, 7%–9%, and >9% comprised 26.6%, 33.0%, 32.2%, and 8.2%, respectively. Among the stage 3–4 and stage 5 CKD groups, the participants with higher HbA1c levels were more likely to have higher hemoglobin levels, and UPCR (all *P* for trend <0.05). Furthermore, patients with a higher HbA1c level exhibited higher levels of blood glucose, lipids (total cholesterol and triglyceride), and inflammatory markers (uric acid and CRP) (all *P* for trend <0.05 except for CRP in stage 5 CKD patients). The participants with higher HbA1c levels in neither group exhibited a higher percentage of CV disease but exhibited a mildly lower percentage of hypertension.

Over the approximately 3-year median follow-up period, 312 (20.0%) and 583 (69.1%) cases of RRT were identified among the patients with stages 3–4 and stage 5 CKD, respectively. The patients with higher HbA1c levels exhibited a higher rate of RRT in stage 3–4 CKD and a more rapid annual eGFR decline was observed in stages 3–5. Incident cases of all-cause mortality were 271 (17.4%) and 259 (30.7%) in the stage 3–4 and stage 5 CKD groups, respectively. Hypoglycemic cases requiring medical assistance occurred were 49 (3.1%) and 37 (4.3%) in the stage 3–4 and stage 5 CKD groups, respectively. The participants with higher HbA1c levels did not exhibit a significantly higher rate of all-cause mortality in either group.

### Multivariate Linear Regression for HbA1c

**Figure 1:**
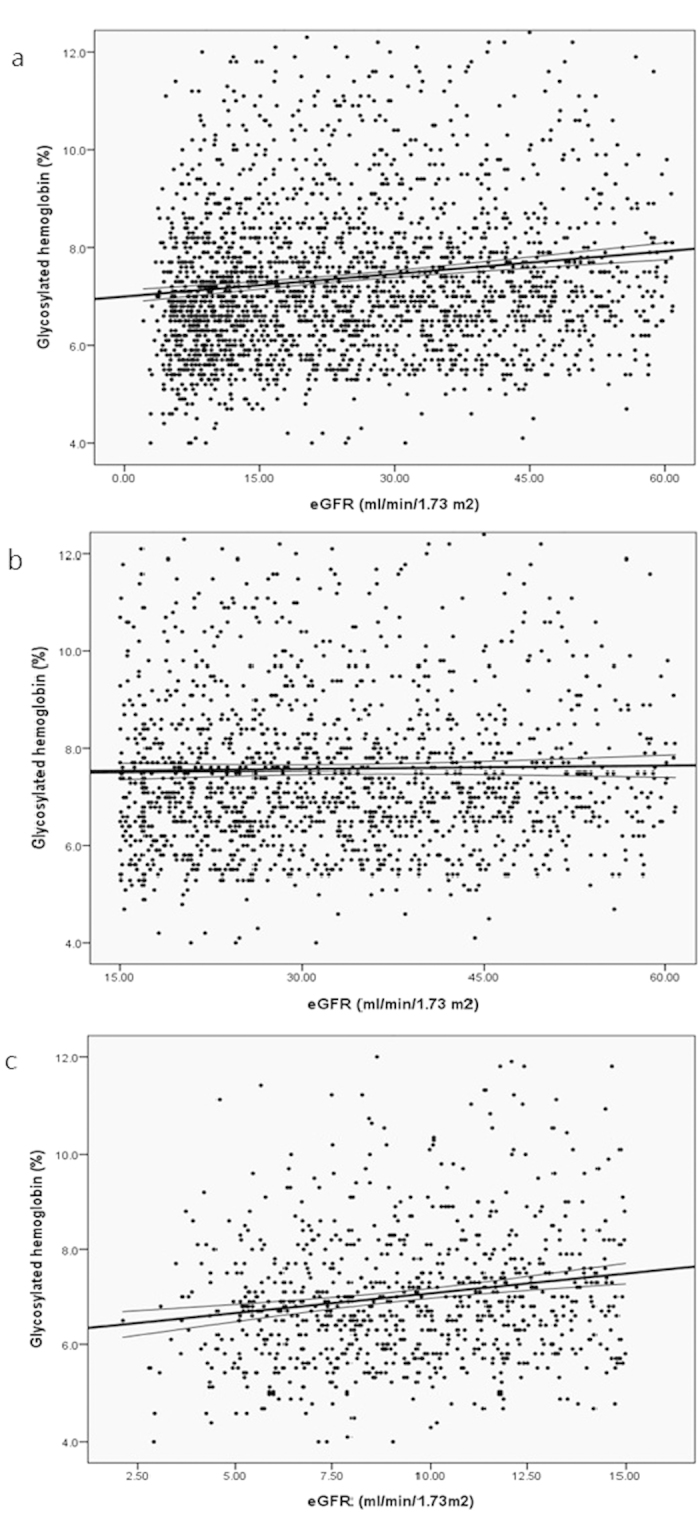
Regression figure for HbA1c and eGFR among subjects with (**a**) all patients (**b**) stage 3-4 and (**c**) stage 5 CKD.

Table 2 shows the results of multivariate linear regression for HbA1c level. In all participants, age, the eGFR, the log UPCR, albumin, hemoglobin, log cholesterol, and log CRP were associated with the baseline HbA1c level. In the subgroup analysis, higher HbA1c level correlated with higher eGFR in patients with stage 5 CKD (95% confidence interval [95% CI], 0.031 to 0.108, *P* < 0.001) but not in patients with stage 3–4 CKD (95% CI, −0.005 to 0.012, *P* = 0.451) (Table 2 and Fig. 1). By contrast, a significant correlation existed between a higher HbA1c level and a poorer UPCR in patients with stage 3–4 CKD, but the correlation was nonsignificant in patients with stage 5 CKD (Table 2 and Fig. 1).

### HbA1c and Clinical Outcome Associations

In patients with stage 3–4 CKD (Table 3), fully adjusted multivariate Cox regression analysis revealed that HbA1c levels of 6%–7%, 7%–9%, and >9% were associated with an increased risk of receiving RRT, with HRs of 1.2 (95% CI, 0.80 to 1.80, *P* = 0.37), 1.38 (95% CI, 0.96 to 1.99, *P* = 0.08), and 1.6 (95% CI, 1.07 to 2.38, *P* = 0.02) (*P* for trend = 0.11), respectively, compared with levels <6%. Moreover, HbA1c levels of 6%–7%, 7%–9%, and >9% were associated with increased risks of all-cause mortality, with HRs of 1.46 (95% CI, 0.96 to 2.21, *P* = 0.07), 1.35 (95% CI, 0.91 to 2.02, *P* = 0.13), and 1.52 (95% CI, 0.97 to 2.38, *P* = 0.07) (*P* for trend = 0.27), respectively, although this association did not reach statistical significance. Similarly, higher HbA1c had a tendency toward a higher risk of CV events combined with all-cause mortality, with HbA1c levels of 6%–7%, 7%–9%, and >9% exhibiting HRs of 1.25 (95% CI, 0.89 to 1.76, *P* = 0.20), 1.29 (95% CI, 0.93 to 1.77, *P* = 0.12), and 1.46 (95% CI, 1.02 to 2.09, *P* = 0.04) (*P* for trend = 0.22), respectively. However, in patients with stage 5 CKD (Table 3), no relationship between the HbA1c levels and clinical outcomes was observed. In addition, a HbA1c level of 7%–9% was associated with a higher likelihood of RRT, with a HR of 1.28 (95% CI, 1.01 to 1.63, *P* = 0.04), whereas the risk of RRT was lower for a HbA1c level >9%, with an HR of 1.01 (95% CI, 0.70 to 1.45, *P* = 0.97), compared with a level <6%. Similarly, higher all-cause mortality was observed for a HbA1c level of 7%–9%, but this rate was lower for a HbA1c level >9%.

**Figure 2:**
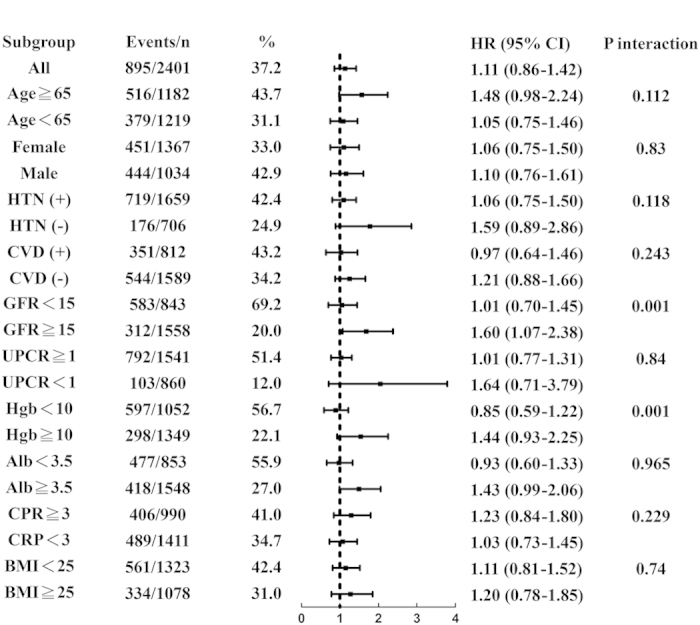
Subgroup analysis of risk of RRT.

Figure 2 presents subgroup analysis results regarding the adjusted risks of RRT. Both the eGFR and hemoglobin affected the association between the HbA1c level and RRT (both *P* for interaction = 0.001); a HbA1c level >9% was associated with a higher risk (HR, 1.44; 95% CI, 0.93 to 2.25) of RRT in patients with hemoglobin >10 mg/dl whereas HbA1c >9% was not associated with a higher risk (HR, 0.85; 95% CI, 0.59 to 1.22) in patients with hemoglobin <10 mg/dl.

## Discussion

In the diabetic patients with stage 3–4 CKD, our study identified that the baseline HbA1c >9% is correlated with higher risks for multiple relevant outcomes, including RRT and combined CV events with all-cause mortality. By contrast, the relationship between HbA1c and clinical outcomes was not significant for those with stage 5 CKD. Accordingly, these findings demonstrate that the predictive value of HbA1c level is stronger at earlier CKD stages.

Glycemic control has been clarified in previous studies as being related to microvascular complications. Chronic hyperglycemia promotes advanced glycation end-product formation, which can increase growth factor production and consequently contribute to extracellular protein deposition, mesangial expansion, gradual glomerular scelrosis, thereby reducing GFR^15^. In a 3-year cohort study, diabetic participants with preserved renal function whose baseline HbA1c exceeded 6% exhibited accelerated eGFR decline^16^. Recently, a meta-analysis of 7 randomized controlled trials (RCTs) of intensive glycemic therapy, defined by lower HbA1c, versus the standard regimen for type 2 DM, reported a significant reduction in microalbuminuria and macroalbuminuria occurrences, but the benefits were inconclusive concerning the effect on clinical renal outcomes, defined by the doubling of the SCr level or ESRD^17^.

Little evidence is available regarding the relationship between HbA1c levels and clinical outcomes in patients with advanced CKD. Se Won Oh *et al*. enrolled a 5-year cohort of 799 patients with DM and an eGFR <60 ml/min/1.73m^2^ and reported that patients with a baseline HbA1c of <6.5% had reduced a risk for ESRD by comparing those with a HbA1c of >6.5%^18^. In people requiring chronic hemodialysis, Oomichi T *et al*. found poor glycemic control is an independent predictor of survival from an observational study in which 114 diabetic CKD patients were enrolled^19^. However, others reported that the CKD stages could influence the association between HbA1c and renal outcomes. A population-based cohort study on patients with DM and stage 3–4 CKD revealed that a baseline HbA1c higher than 7% was strongly associated with an increased risk of ESRD. Moreover, the magnitude of increased risk with higher HbA1c levels seemed attenuate in patients with stage 4 CKD compared with patients with stage 3 CKD^8^. Considering the rate of eGFR decline, a recent cohort study in Taiwan demonstrated that for patients with higher preceding HbA1c levels, the negative effects on eGFR deterioration were stronger at stage 3–4 CKD than stage 1–2 or stage 5, but the outcomes of ESRD were not reported^20^. Our results were consistent with these data. We observed a HbA1c >9%, comparing with a HbA1c <6%, was associated with an increased risk for ESRD in the stage 3–4 CKD group. Conversely, a corresponding trend was not observed in patients with stage 5 CKD. Our study is the first to recruit a large-scale sample of patients with stage 5 CKD and, according to this finding, we conclude that HbA1c could not be sufficient predict ESRD in patients with stage 5 CKD.

The prognostic role of HbA1c in patients with stage 5 CKD was unclear because impaired glucose metabolism in advanced CKD, and the HbA1c level may be altered by anemia, or erythropoiesis-stimulating agent use. First, it is well-known that a marked reduction in insulin clearance can occur until the GFR falls to less than 15–20 ml/min^21^. Agarwal *et al*. demonstrated that glycemic control, as assessed by random blood glucose, improved in patients with late-stage CKD^22^. Our data found that a lower HbA1c level was correlated with a lower eGFR in patients with stage 5 CKD but not in those with stage 3–4 CKD, which could be partly explained by hyperinsulinemia affecting the HbA1c level in stage 5 CKD. Second, glycated hemoglobin formation is reduced in patients with CKD because the fragile red blood cell (RBC) has shortened lifespan by 30%–70%, and carbamylated hemoglobin molecules in the uremic environment become resistant to glycosylation^23^,^24^. Administering erythropoietin stimulating agents (ESAs) to patients with anemia also augments, in peripheral blood, the proportion of young RBCs, which have a lower rate of glycosylation than do old RBCs, thereby altering glycosylated hemoglobin formation^25^. The results of some studies support this notion. Agarwal *et al*. reported that, among 128 patients with DM and stage 1–5 CKD, a decline in HbA1c was correlated with CKD stages, but this relationship disappeared after adjustment for hemoglobin^22^. In addition, Freedman *et al*. confirmed, in diabetic patients with stage 3–4 CKD, an inverse correlation between the eGFR and the glucose/HbA1c ratio, which indicated that HbA1c could be falsely low in lower eGFR^26^. Accordingly, HbA1c levels appear to be falsely low in subjects with DM and advanced CKD^27^,^28^,^29^. Our data again confirm the positive correlation between HbA1c and Hb in stage 3–5 CKD, but the positive correlation between HbA1c and eGFR only exists in stage 5 CKD. HbA1c level may not accurately indicate glycemic control during the deterioration of kidney function, and based on our study it is less prognostic in stage 5 CKD.

Measuring HbA1c levels earlier might increase their prognostic value. The term “legacy effect” has been used in some RCTs to describe the ongoing benefits of better glycemic control even after intervention ceases. A proposed mechanism for this is that fewer advanced glycation end products confer long-term protection. In the Epidemiology of Diabetes Interventions and Complications trial, participants who were originally assigned to an intensive control group continued exhibiting benefits in sustainable reductions of macroalbuminuria and renal function impairment during the 8-year post-trial period, even though the average HbA1c levels of the intensive group and conventional groups lost their difference (8.0% and 8.2%)^30^,^31^,^32^. This phenomenon implies that HbA1c measurement in late CKD stages may be too late to determine their long term glycemic control. Our observational study identified no increase in the risks of ESRD and the composite endpoint of CV events and all-cause mortality in stage 5 CKD, even when the baseline HbA1c level was >9%. In other words, the HbAlc level is more useful in stage 3–4 CKD than it is in stage 5 CKD in predicting clinical prognosis, either because of multiple factors influencing HbA1c production in stage 5 CKD or the possible legacy effect.

Regarding patients with CKD, attempting to control HbA1c levels as low as possible is controversial if we take safety into account because doing so increases the risk of hypoglycemia. This is on account of prolonged half-life of antidiabetic drugs and reduced renal insulin clearance, degradation of insulin in peripheral tissues, glycogen stores, and renal gluconeogenesis^33^. Furthermore, the CV mechanism of hypoglycemia exists in sympathoadrenal stimulation and the inflammation reaction, which may bring about endothelial dysfunction and coagulation abnormalities, eventually causing QT prolongation, cardiac arrhythmia, and eventual CV events^34^. Several RCTs have attempted to lower HbA1c levels aggressively in diabetic patients with preserved kidney function, which illustrated the inconsistent findings regarding CV benefits and mortality, and the rates of hypoglycemia were greater in the intensive therapy groups^4^,^5^,^35^. In addition, one meta-analysis of 5 RCTs involving patients with type 2 DM also suggested that intensive glucose therapy reduced nonfatal myocardial infarction and coronary heart disease but not all-cause mortality^7^. These data told that the adverse sequelae of hypoglycemia might partly cause the inconsistent CV and mortality outcomes. Moreover, a large-scale UK observational study reported a general U-shaped association of the mean HbA1c level with all-cause mortality and CV events, with the HbA1c threshold at approximately 7.5% and higher or lower levels related to increased risks^36^. Nevertheless, our cohort did not demonstrate a U-shape association, which seems not in concordance with prior studies. This discrepancy could be interpreted by the low incidence of hypoglycemia in our patients, who were provided with behavioral instruction to prevent hypoglycemia. Furthermore, patients with low HbA1c level in an observational study do not parallel to those in a clinical trial receiving strict glycemic control. These patients with low HbA1c level in our study group were not forced to receive intensive intervention and may have less severe diabetes. Thus, hypoglycemia and CV disadvantages did not emerge.

There are several limitations to this study. First, we relied on the baseline HbA1c rather than the mean HbA1c level. HbA1c level at one point could not reflect the actual glucose control during the follow-up period. However, because of the gradual decline of HbA1c as CKD stage progresses and the possible legacy effect, we considered baseline HbA1c could serve a reasonable indicator when CKD stage was classified at the same time. Second, we did not measure serial blood glucose levels which may represent actual glucose control better than the HbA1c values, and thus we could not analyze the relationship between the CKD stages with variation of blood glucose. Third, it is our limitation to use immunoassays in the hospital as HbA1c testing methodology since immunological methods for the detection of HbA1c is more reliable in uremic environment. Fourth, we did not have data on medications that the patients used to control their DM, such as oral hypoglycemic agents and insulin. In addition, we did not collect the information on the ESAs doses or other medications that may alter RBC production; some studies have found that the ESA dose is inversely related to HbA1c level^37^. Therefore, we could not adjust for these potential confounders. Fourth, this was a cohort study that cannot evaluate the clinical effects of intensive intervention on glucose control in patients with advanced CKD.

In conclusion, an HbA1c >9% predicts an increased risk for ESRD, and composite outcome of CV event and mortality in patients with stage 3–4 CKD. By contrast, in stage 5 CKD, predictive value of HbA1c level is weaker. Accordingly, poor glycemic control is still associated with poor clinical outcomes in diabetic patients with stage 3–4 CKD. Whether glycemic control itself is associated with clinical outcomes in diabetic patients with stage 5 CKD or not, it needs further study.

## Additional Information

**How to cite this article**: Kuo, I.-C. *et al*. Glycated Hemoglobin and Outcomes in Patients with Advanced Diabetic Chronic Kidney Disease. *Sci. Rep.* **6**, 20028; doi: 10.1038/srep20028 (2016).

## Figures and Tables

**Table 1:** Baseline Characteristics of stage 3-4 and stage 5 CKD DM subjects by HbA1c level.

**Variables**	**All**	**CKD stage 3-4**					**CKD stage 5**				
		**HbA1c level (%)**					**HbA1c level (%)**				
		**<6**	**6-7**	**7–9**	**>9**	***P for trend***	**<6**	**6–7**	**7–9**	**>9**	***P for trend***
**No. of patients**	2401	263	447	590	258	**—**	224	279	271	69	**—**
**Demographics and Medical History**											
Age (years)	64.3 ± 12.6	67.1 ± 12.7	64.7 ± 13.3	64.8 ± 12.8	61.8 ± 11.9	<0.001	63.7 ± 13.1	64.5 ± 11.7	63.0 ± 12.0	61.1 ± 11.9	0.232
Male (n[%])	1034 (43%)	109 (41.4%)	162 (36.2%)	225 (38.1%)	93 (36.0%)	0.371	123 (54.9%)	137 (49.1%)	142 (52.4%)	43 (62.3%)	0.387
Hypertension (n[%])	1695 (71%)	193 (73.4%)	299 (66.9%)	366 (62.0%)	167 (64.7%)	0.030	193 (86.2%)	221 (79.2%)	203 (74.9%)	53 (76.8%)	0.011
CVD (n[%])	812 (33.8%)	92 (35.0%)	148 (33.1%)	159 (26.9%)	87 (33.7%)	0.496	84(37.5%)	107 (38.4%)	105 (38.7%)	30 (43.5%)	0.416
IHD (n[%])	462 (19.2%)	39 (14.8%)	87 (19.5%)	96 (16.3%)	52 (20.2%)	0.298	54 (24.1%)	69 (24.7%)	47 (17.3%)	18 (26.1%)	0.472
CHF (n[%])	406 (16.9%)	35 (13.3%)	71 (15.9%)	76 (12.9%)	38 (14.7%)	0.978	47 (21.0%)	65 (23.3%)	55 (20.3%)	19 (27.5%)	0.503
CBVD (n[%])	492 (20.5%)	70 (26.6%)	95 (21.3%)	99 (16.8%)	55 (21.3%)	0.111	48 (21.4%)	53 (19.0%)	55 (20.3%)	17 (24.6%)	0.652
BMI (m^2^/kg)	24.9 ± 3.9	24.1 ± 3.5	25.5 ± 4.2	25.4 ± 3.9	25.2 ± 3.6	<0.001	24.3 ± 3.9	24.9 ± 4.2	24.2 ± 3.7	25.0 ± 3.5	<0.001
MAP (mmHg)	99.9 ± 14.2	98.1 ± 13.7	98.3 ± 13.7	99.7 ± 13.7	101.5 ± 15.4	<0.001	101.3 ± 14.7	101.3 ± 14.7	100.6 ± 14.2	100.0 ± 15.0	<0.001
**Laboratory Data**											
eGFR (ml/min/1.73 m^2^)	24.7 ± 14.9	31.5 ± 11.3	33.5 ± 12.1	33.6 ± 12.1	32.6 ± 11.9	<0.001	9.0 ± 3.0	9.1 ± 2.9	9.8 ± 2.9	10.2 ± 2.9	<0.001
UPCR (mg/g)	1738 (547 to 4547)	782 (282 to 3090)	820 (252 to 2241)	1320 (330 to 3736)	1997 (570 to 4544)	<0.001	2830 (1259 to 5994)	2818 (1609 to 6026)	3498 (1513 to 7304)	4266 (1899 to 7552)	0.012
Albumin (g/dl)	3.7 ± 0.6	3.8 ± 0.6	3.9 ± 0.6	3.7 ± 0.6	3.6 ± 0.5	0.731	3.5 ± 0.6	3.5 ± 0.5	3.5 ± 0.6	3.3 ± 0.5	0.491
Hemoglobin (g/dl)	10.7 ± 2.3	11.2 ± 2.4	11.7 ± 2.2	11.6 ± 2.2	11.8 ± 2.0	<0.001	8.7 ± 1.3	9.2 ± 1.4	9.4 ± 1.5	9.3 ± 1.6	0.013
Blood glucose (mg/dl)	130.6 ± 55.2	101.2 ± 23.6	119.9 ± 42.3	139.8 ± 51.9	183.2 ± 74.1	<0.001	100.7 ± 30.5	114.5 ± 36.1	136.6 ± 58.5	174.2 ± 78.3	<0.001
HbA1c (%)	7.4 ± 1.7	5.6 ± 0.4	6.6 ± 0.3	7.8 ± 0.5	10.7 ± 1.4	<0.001	5.5 ± 0.4	6.6 ± 0.3	7.8 ± 0.6	10.6 ± 1.5	<0.001
Total calcium (mg/dl)	9.1 ± 0.8	9.2 ± 0.6	9.3 ± 0.6	9.2 ± 0.7	9.3 ± 0.7	<0.001	8.7 ± 1.0	8.8 ± 0.9	8.8 ± 0.8	8.7 ± 0.8	0.537
Phosphate (mg/dl)	4.4 ± 1.2	4.0 ± 0.7	4.0 ± 0.9	4.0 ± 0.9	4.1 ± 0.9	0.012	5.3 ± 1.3	5.2 ± 1.2	5.2 ± 1.3	5.2 ± 1.3	0.853
Uric acid (mg/dl)	8.0 ± 2.0	7.8 ± 1.8	8.0 ± 1.9	7.5 ± 1.7	7.9 ± 2.4	<0.001	8.0 ± 2.0	8.2 ± 1.9	8.8 ± 2.0	8.6 ± 1.9	<0.001
Total cholesterol (mg/dl)	192 (161 to 225)	182 (157 to 214)	188 (161 to 219)	196 (165 to 225)	212 (174 to 254)	<0.001	182 (150 to 212)	191 (152 to 222)	197 (166 to 232)	196 (170 to 238)	<0.001
Triglyceride (mg/dl)	139 (99 to 205)	118 (83 to 174)	132 (98 to 199)	142 (100 to 201)	194 (132 to 272)	<0.001	123 (93 to 173)	130 (95 to 189)	153 (102 to 220)	174 (128 to 274)	<0.001
CRP (mg/l)	1.9 (0.5 to 7.9)	1.0 (0.3 to 5.7)	1.4 (0.4 to 6.3)	1.6 (0.4 to 7.0)	2.4 (0.4 to 14.3)	0.009	2.3 (0.5 to 8.8)	3.0 (0.5 to 10.7)	3.0 (0.5 to 10.1)	2.1 (0.5 to 6.5)	0.988
**Clinical outcomes**											
Follow-up days	993 (539 to 1617)	959 (588 to 1605)	1036 (585 to 1635)	1016 (581 to 1638)	1181 (779 to 1729)	0.036	812 (394 to 1437)	939 (399 to 1617)	938 (451 to 1610)	862 (364 to 1600)	0.161
Annual eGFR decline (ml/min/1.73 m^2^/year)	−3.1 (−7.7 to −0.5)	−2.3 (−6.7 to 0.2)	−2.0 (−5.8 to 1.2)	−3.0 (−7.9 to −0.2)	−4.7 (−11.1 to −0.8)	<0.001	−3.1 (−6.6 to −1.1)	−3.6 (−7.8 to −1.3)	−4.1 (−9.1 to −1.6)	−5.2 (−8.6 to −2.4)	0.006
Rapid eGFR decline^a^	898 (37.4%)	92 (35.0%)	125 (28.0%)	220 (37.3%)	126 (48.8%)	<0.001	75 (33.5%)	107 (38.4%)	118 (43.5%)	35 (50.7%)	0.003
RRT	895 (37.3%)	50 (19.0%)	68 (15.2%)	119 (20.1%)	75 (29.1%)	<0.001	155 (69.2%)	195 (69.9%)	186 (68.6%)	47 (68.1%)	0.354
Hypoglycemia	86 (3.5%)	9 (3.4%)	14 (3.1%)	19 (3.2%)	7 (2.7)	0.436	10 (4.5%)	12 (4.3%)	12 (4.4%)	3 (4.3%)	0.741
All-cause mortality	530 (22.1%)	43 (16.3%)	69 (15.4%)	105 (17.8%)	54 (20.9%)	0.080	59 (26.3%)	86 (30.8%)	94 (34.7%)	20 (29.0%)	0.254
CV event	490 (20.4%)	47 (17.9%)	67 (15.0%)	100 (16.9%)	56 (21.7%)	<0.001	57 (25.4%)	75 (26.9%)	68 (25.1%)	20 (29.0%)	0.269
CV event + all-cause mortality	754 (31.4%)	63 (24.0%)	96 (21.5%)	145 (24.5%)	86 (33.3%)	<0.001	91 (40.6%)	117 (42.0%)	123 (45.4%)	33 (47.8%)	0.317

Data presented as mean ± standard deviation, median (interquartile range) or count (percentage) unless otherwise noted.

Abbreviations: CKD, chronic kidney disease; DM, diabetes mellitus; HbA1c, glycated hemoglobin; CVD, cardiovascular disease; IHD, ischemic heart disease; CHF, congestive heart failure; CBVD, cerebral vascular disease; BMI, body mass index; MAP, mean arterial pressure; eGFR, estimated glomerular filtration rate; UPCR, urine protein-to-creatinine ratio; CRP, C-reactive protein.

*P* < 0.05 indicates a significant difference among the four HbAlc groups.

Abbreviations: CKD, chronic kidney disease; eGFR, estimated glomerular filtration rate; RRT, renal replacement therapy.

*P* < 0.05 indicates a significant difference among the four HbAlc groups.

^a^Annual eGFR decline more than -5 ml/min/1.73 m^2^/year

**Table 2:** Multivariate linear regression of HbA1c level.

**Variables**	**All**		**CKD stage 3-4**		**CKD stage 5**	
	**β(95% CI)**	***p*****-value**	**β(95% CI)**	***p*****-value**	**β(95% CI)**	***p*****-value**
Age (years)	−0.009 (−0.014 to −0.003)	0.002	−0.009 (−0.016 to −0.002)	0.010	−0.012 (−0.021 to −0.003)	0.010
Male vs.female	0.029 (−0.116 to 0.174)	0.693	−0.060 (−0.254 to 0.134)	0.546	0.116 (−0.097 to 0.329)	0.283
CVD	0.030 (−0.113 to 0.173)	0.680	0.015 (−0.174 to 0.205)	0.876	0.061 (−0.150 to 0.272)	0.573
BMI (m^2^/kg)	0.002 (−0.015 to 0.002)	0.795	0.005 (−0.018 to 0.028)	0.684	−0.005 (−0.031 to 0.022)	0.738
MAP (mmHg)	−0.002 (−0.007 to 0.003)	0.421	0.004 (−0.003 to 0.010)	0.285	−0.009 (−0.017 to−0.002)	0.011
eGFR (ml/min/1.73 m^2^)	0.012 (0.005 to 0.018)	<0.001	0.003 (−0.005 to 0.012)	0.451	0.070 (0.031 to 0.108)	<0.001
Log (UPCR)	0.308 (0.146 to 0.469)	<0.001	0.254 (0.060 to 0.448)	0.010	0.233 (−0.075 to 0.541)	0.138
Albumin (g/dl)	−0.210 (−0.343 to −0.077)	0.002	−0.206 (−0.382 to −0.029)	0.022	−0.199 (−0.397 to −0.001)	0.049
Hemoglobin (g/dl)	0.099 (0.058 to 0.141)	<0.001	0.072 (0.021 to 0.123)	0.006	0.152 (0.076 to 0.228)	<0.001
Log (cholesterol)	2.098 (1.524 to 2.672)	<0.001	2.142 (1.391 to 2.892)	<0.001	1.747 (0.871 to 2.623)	<0.001
Log (CRP)	0.059 (−0.011 to 0.130)	0.097	0.079 (−0.011 to 0.168)	0.085	0.024 (−0.088 to 0.135)	0.677
Phosphate (mg/dl)	−0.063 (−0.133 to 0.006)	0.072	0.017 (−0.092 to 0.127)	0.757	−0.010 (−0.103 to 0.082)	0.832

Abbreviations: HbA1c, glycated hemoglobin; CKD, chronic kidney disease; CVD, cardiovascular disease; BMI, body mass index; MAP, mean arterial pressure; eGFR, estimated glomerular filtration rate; UPCR, urine protein-to-creatinine ratio; CRP, C-reactive protein.

*P* < 0.05 indicated a significantly associated with HbA1c levels.

**Table 3:** Risk of outcomes among subjects with stage 3-4 and stage 5 CKD by HbA1c level.

	**All**				**CKD stage 3-4**				**CKD stage 5**			
	**HbA1c level (%)**				**HbA1c level (%)**				**HbA1c level (%)**			
	<**6**	**6–7**	**7–9**	**>9**	<**6**	**6–7**	**7–9**	**>9**	<**6**	**6–7**	**7–9**	**>9**
**HR (95% CI) for RRT**												
Unadjusted	1	0.79 (0.65–0.96)*	0.79 (0.65–0.95)*	0.64 (0.51–0.82)**	1	0.76 (0.51–1.13)	1.12 (0.79–1.60)	1.30 (0.89–1.91)	1	1.15 (0.92–1.45)	1.10 (0.88–1.38)	0.92 (0.64–1.31)
Adjusted^a^	1	1.16 (0.95–1.41)	1.22 (1.01–1.49)*	1.11 (0.86–1.42)	1	1.20 (0.80–1.80)	1.38 (0.96–1.99)	1.60 (1.07–2.38)*	1	1.14 (0.90–1.44)	1.28 (1.01–1.63)*	1.01 (0.70–1.45)
**HR (95% CI) for all-cause mortality**												
Unadjusted	1	1.05 (0.80–1.38)	1.10 (0.85–1.43)	0.92 (0.66–1.27)	1	1.11 (0.74–1.68)	1.21 (0.82–1.79)	1.22 (0.79–1.88)	1	1.12 (0.78–1.62)	1.24 (0.87–1.77)	0.92 (0.51–1.67)
Adjusted^a^	1	1.26 (0.96–1.66)	1.29 (0.99–1.68)	1.30 (0.93–1.83)	1	1.46 (0.96–2.21)	1.35 (0.91–2.02)	1.52 (0.97–2.38)	1	1.09 (0.75–1.57)	1.16 (0.80–1.67)	0.97 (0.53–1.79)
**HR (95% CI) for CV event**												
Unadjusted	1	0.86 (0.66–1.12)	0.85 (0.66–1.09)	0.86 (0.63–1.17)	1	0.83 (0.56–1.22)	0.99 (0.69–1.41)	1.01 (0.67–1.52)	1	1.02 (0.71–1.46)	0.86 (0.59–1.25)	1.12 (0.65–1.91)
Adjusted^a^	1	0.99 (0.76–1.29)	0.97 (0.74–1.25)	1.11 (0.80–1.54)	1	1.02 (0.69–1.51)	1.14 (0.79–1.64)	1.16 (0.76–1.76)	1	1.04 (0.72–1.50)	0.82 (0.56–1.22)	1.13 (0.64–1.98)
**HR (95% CI) for CV event + all cause mortality**												
Unadjusted	1	0.88 (0.71–1.10)	0.92 (0.74–1.14)	0.91 (0.70–1.18)	1	0.96 (0.68–1.34)	1.10 (0.80–1.50)	1.23 (0.87–1.75)	1	0.94 (0.70–1.27)	0.98 (0.73–1.31)	1.04 (0.67–1.62)
Adjusted^a^	1	1.04 (0.84–1.31)	1.06 (0.85–1.31)	1.25 (0.95–1.63)	1	1.25 (0.89–1.76)	1.29 (0.93–1.77)	1.46 (1.02–2.09)*	1	0.93 (0.69–1.25)	0.91 (0.67–1.23)	1.07 (0.68–1.69)

^a^The Cox proportional hazard model was adjusted for age, gender, estimated glomerular filtration rate, log (urine protein-to-creatinine ratio), cardiovascular disease, mean blood pressure, hemoglobin, albumin, log (cholesterol), log (C-reactive protein), phosphorus and body mass index.

*p < 0.05 and **p < 0.001 indicate significantly different with reference group.
